# Impact of Reduced Vitamin D Levels on Pain, Function, and Severity in Knee or Hip Osteoarthritis

**DOI:** 10.3390/nu17030447

**Published:** 2025-01-26

**Authors:** Cláudia Nascimento Montemor, Marcos Tadeu Parron Fernandes, Audrey Souza Marquez, Paulo Roberto Bignardi, Regina Célia Poli, Gustavo Aliano Gâmbaro, Rubens Alexandre da Silva, Suzy Ngomo, Karen Barros Parron Fernandes

**Affiliations:** 1 School of Medicine, Pontifical Catholic University of Parana (PUCPR), Londrina 86072-360, PR, Brazil; claudia.montemor@pucpr.br (C.N.M.); paulo.bignardi@pucpr.br (P.R.B.); gustavoa.gambaro@pucpr.br (G.A.G.); 2 Social Responsability Institute, Sirio Libanes Hospital, Sao Paulo 01401-000, SP, Brazil; mparron@yahoo.com.br; 3 Department of Research and Scientific Advisory, Integrative Health Technologies LLC (IHT), Orlando, FL 3885-3569, USA; audreymarquez@icloud.com; 4 Health Sciences Research Center, University Pitágoras Unopar (UNOPAR), Londrina 86047-790, PR, Brazil; regina.frederico@cogna.com.br; 5 Department of Health Sciences, Université du Québec à Chicoutimi (UQAC), Saguenay, QC G7H 2B1, Canada; rdsilva@uqac.ca (R.A.d.S.); sngomo@uqac.ca (S.N.); 6 Graduate Program in Health Sciences (PPGCS), School of Medicine, Pontifical Catholic University of Parana (PUCPR), Curitiba 80215-901, PR, Brazil

**Keywords:** osteoarthritis, elderly, vitamin D, functional status, severity, tumor necrosis factor, interleukin-6

## Abstract

Background: Vitamin D is beneficial for musculoskeletal health. Although low levels of vitamin D are linked to increased pain in knee osteoarthritis (OA), their association with functionality remains understudied. Objective: This study aimed to investigate the association between vitamin D deficiency and functional status in elderly individuals with OA and explore the potential correlation between vitamin D deficiency and plasma levels of tumor necrosis factor alpha (TNF-α) and interleukin-6 (IL-6). Methods: The study included older adults (≥60 years) from an ageing study, encompassing 105 OA patients and 152 controls. OA diagnosis was confirmed radiographically, and the WOMAC questionnaire assessed functional impairment in these patients. Blood samples were collected to measure 25(OH) vitamin D levels by chemiluminescence and TNF-α and IL-6 levels by ELISA. Results: Patients with vitamin D insufficiency/deficiency exhibited more severe cases of OA compared to those with normal vitamin D levels (*p* = 0.04). Vitamin D levels were inversely correlated with functional impairment in OA, according to the WOMAC Index (global: rS = −0.25, *p* = 0.01; pain: rS = −0.21, *p* = 0.03). Moreover, OA patients with vitamin D deficiency showed significantly higher levels of TNF-α and IL-6 (*p* < 0.05, Mann−Whitney test). Conclusions: Reduced levels of vitamin D are associated with more severe cases of hip and knee osteoarthritis, increased pain, greater functional impairment, and elevated serum levels of TNF-α and IL-6. Further research is required to elucidate the mechanisms underlying the influence of vitamin D on osteoarthritis and to evaluate the potential benefits of vitamin D supplementation for mitigating disease symptoms and progression.

## 1. Introduction

Osteoarthritis (OA) is a musculoskeletal disorder with a complex and multifactorial pathogenesis. It is a leading cause of pain and disability worldwide, significantly impairing health-related quality of life [1,2]. OA is estimated to affect over 7% of the global population, with projections suggesting an increase in its prevalence by 60 to 100% by the year 2050 [3,4].

Clinically, OA most commonly affects the knee and hip joints, leading to symptoms such as swelling, pain, and reduced muscle strength [5]. The onset of OA is influenced by a variety of factors, including genetic predisposition, biomechanical stresses, and alterations in the articular joint or synovial membrane. Key risk factors include obesity, excessive joint stress, trauma, periarticular injuries, and occupational factors [4,6].

There is growing evidence indicating that adequate levels of vitamin D are beneficial for several physiological functions, such as musculoskeletal health [7,8] and cartilage and bone metabolism [9]. In contrast, vitamin D deficiency has been associated with reduced expression of vitamin D receptors in skeletal muscles, leading to decreased muscle mass, especially in the elderly [8].

Low levels of vitamin D have been linked to musculoskeletal disorders, such as temporomandibular disorders [10]. Indeed, they have also been linked to increased knee OA pain, particularly in men [11,12]. Recently, a systematic review by Georgescu et al. highlighted significant variability in the data regarding this association and underscored the need for more robust evidence to clarify the relationship [13]. However, the exact mechanism underlying the association of vitamin D with OA onset and progression has not been fully demonstrated.

It is noteworthy that elevated levels of inflammatory biomarkers, such as interleukin-6 (IL-6) and tumor necrosis factor alpha (TNF-α), have been observed in patients with both knee osteoarthritis (KOA) and vitamin D deficiency [14,15]. 

Although inflammatory cytokines may mediate the effects of vitamin D in OA, this relationship has not been conclusively demonstrated. Therefore, the aim of this study was to investigate the association between vitamin D deficiency and disease severity and functional status in patients with OA. Additionally, we sought to determine whether TNF-α and IL-6 could play a role as mediators of the detrimental effects of vitamin D deficiency in knee and hip osteoarthritis.

## 2. Methods

### 2.1. Ethical Procedures

The research protocol was approved by the University Research Ethics Committee (Protocol # 090-07). Prior to any procedures, all participants were thoroughly informed about the nature of the study. They were required to provide written, voluntary informed consent to confirm their agreement to participate.

### 2.2. Study Design and Sample Size

Participants were recruited from a cohort study on ageing and longevity in Londrina, Paraná, Brazil (EELO project, an abbreviation of the Portuguese title). The EELO study aimed to evaluate socio-demographic factors and health indicators in older adults. From a population of 43,610 elderly individuals living in the urban area of Londrina, 518 subjects were recruited to form a representative sample. Participants were selected from the registers of all community outpatient public health units, and the sample was randomly stratified based on the proportion of older adults in each region of the city. Further details can be found at http://www2.unopar.br/eelo/index.html (accessed on 5 January 2020). From this broad sample, 198 individuals self-reported OA. However, only 105 had confirmed hip or knee OA with no other exclusion criteria. Therefore, this study employed a cross-sectional design and included a convenience sample of 257 elderly individuals (105 from the OA group and 152 matched control subjects).

### 2.3. Eligibility Criteria of the Study Population

To be included in the study, participants had to meet the following criteria: (1) be male or female; (2) be aged over 60 years; (3) have a previous medical diagnosis of or self-reported hip or knee OA (OA group) or be a patient without other inflammatory conditions, matched by gender and age to the OA group (matched control group); and (4) provide voluntary consent and sign the informed consent form. Exclusion criteria included individuals who (1) had full or partial prosthetics in the hip or knee; (2) had a previous diagnosis of other musculoskeletal disorders (e.g., rheumatoid arthritis, fibromyalgia, systemic lupus erythematosus, or other rheumatic diseases) or any inflammatory condition; (3) had post-traumatic or post-septic arthritis; (4) had more than one site affected by OA (such as hip and knee); (5) had skeletal dysplasia; (6) were using corticosteroids, other anti-inflammatory drugs, or immunosuppressive medications; (7) had undergone arthroplasty; or (8) had diabetes.

### 2.4. Osteoarthritis Severity

Participants who self-reported hip or knee osteoarthritis underwent X-ray examinations. All X-rays were performed at the same health facility following standard procedures and were evaluated by the same radiologist physician. These procedures were essential for confirming the diagnosis and determining the severity of OA according to the Kellgren and Lawrence grading scale [16], ranging from Grade 0 (no changes) to Grade IV (severe disease with marked joint space narrowing). Patients were stratified into the mild/moderate (Grade I and II) or severe (Grade III and IV) categories by a general physician afterward.

### 2.5. Functional Evaluation

The Western Ontario and McMaster Universities Osteoarthritis Index (WOMAC), the gold standard for assessing functional status in individuals with osteoarthritis, was used for this evaluation. This tool includes items related to pain, stiffness, and overall function, with higher scores indicating worse condition [17]. A previously translated and validated Portuguese version of the WOMAC was used. The questionnaires were administered through interviews conducted by trained examiners in the morning, prior to the X-ray examination.

### 2.6. Sample Collection and Quantification of 25-Hydroxyvitamin D [25(OH)D]

Serum, fluorinated plasma, and EDTA samples were collected from each participant after a 10-h fasting period. Vitamin D levels were measured using automated chemiluminescence (Architect iSR2000—Abbott© Diagnostics, Lake Forest, IL, USA). This chemiluminescent microparticle immunoassay (CMIA) quantifies 25(OH)D2 and D3 in human serum and plasma within a range of 0 to 160 ng/mL. Vitamin D status was classified based on 25(OH)D according to the Brazilian Consensus: normal (levels higher than 30 ng/mL), insufficiency (levels ranging between 20 to 30 ng/mL), and deficiency (levels lower than 20 ng/mL) [18].

### 2.7. Cytokine Levels

Blood samples were centrifuged to separate the serum, which was stored at −80 °C until further analysis. Serum samples from participants in both the OA and control groups were evaluated for cytokine levels. IL-6 and TNF-α quantification was performed using the enzyme-linked immunosorbent assay (ELISA) method, following the manufacturer’s instructions and standard operating procedures.

### 2.8. Statistical Analysis

A confidence interval of 95% and a significance level of 5% (*p* < 0.05) were set. As the data were not normally distributed (as determined by the Shapiro−Wilk test), descriptive statistics were reported as medians and interquartile ranges. Spearman’s correlation was used to assess the relationship between vitamin D deficiency and functional status. The Mann−Whitney U test was employed to compare serum cytokine levels (TNF-α and IL-6) according to vitamin D status and OA severity, as well as to compare general characteristics between groups. Additionally, the chi-square test was used to analyze the distribution of vitamin D levels between the OA and control groups, explore potential associations between vitamin D and OA severity, and compare gender distributions.

## 3. Results

A total of 257 elderly individuals participated in this study, with 105 in the osteoarthritis (OA) group and 152 in the control group. 

No differences across the groups were observed regarding age (Control: 71.2 ± 6.5 vs. OA group: 73.0 ± 12) and BMI (Control: 28.0 ± 4.7 versus OA group: 28.5 ± 5.3). However, the proportion of females was higher in the OA group (75.9%) than in the control group (59.9%). Similarly, no differences in anthropometric data were shown between OA subgroups. Indeed, the groups were paired according to age, BMI, and gender (Table 1).

The most severe OA was observed in individuals with vitamin D insufficiency or deficiency, with a prevalence of 56.6% (*p* = 0.04; Table 2).

Vitamin D levels were negatively correlated with the global WOMAC (rS = −0.25, *p* = 0.01) and pain (rS = −0.21, *p* = 0.03). However, no significant correlation was observed between vitamin D levels and stiffness or function in this sample (Table 3).

**Figure 1:**
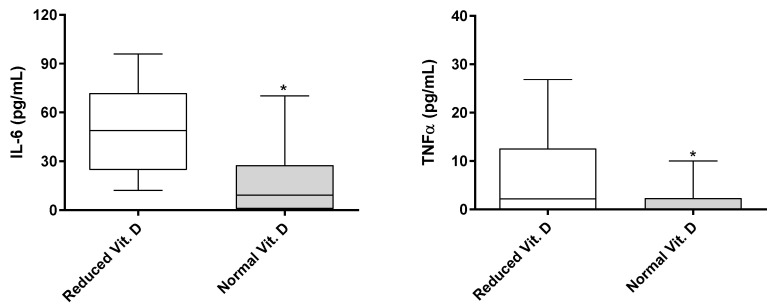
Comparison of IL-6 and TNF-α serum levels (pg/mL) across different vitamin D classifications in patients with hip or knee osteoarthritis. * Statistically significant difference determined by the Mann-Whitney test (*p* < 0.05).

**Figure 2:**
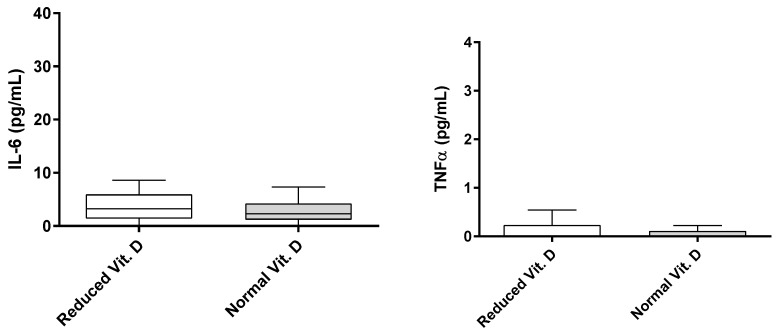
Comparison of IL-6 and TNF-α serum levels (pg/mL) across different vitamin D classifications in individuals without osteoarthritis or other inflammatory diseases.

Furthermore, patients with reduced vitamin D levels exhibited significantly higher serum levels of TNF-α and IL-6, as determined by the Mann−Whitney test (*p* < 0.05, Figure 1). In contrast, serum levels of TNF-α and IL-6 were not associated with vitamin D in control individuals without osteoarthritis or other inflammatory conditions, as shown by the Mann−Whitney test (*p* > 0.05, Figure 2).

## 4. Discussion

This study demonstrates that older adults with reduced levels of vitamin D exhibit more severe OA and increased levels of IL-6 and TNF-α, suggesting that these cytokines could potentially be involved in this mechanism. This result is in agreement with that of Tekali et al. [19], who reported a higher prevalence of vitamin D deficiency in patients with knee OA. As the etiopathogenesis of OA is not completely understood, therapeutic options are not always successful. Therefore, understanding the etiology and identifying and eliminating potential pathogenic factors could make a significant contribution to knowledge about OA.

Additionally, we identified an inverse correlation between vitamin D levels and the Global Index of Functional Status, as measured by the WOMAC scale. Individuals with hypovitaminosis D reported worse global scores and higher pain scores. However, the mechanisms linking low vitamin D levels to pain and joint function remain incompletely understood.

These findings are consistent with those of previous research, highlighting the role of vitamin D in modulating various cell types, including osteoblasts, osteoclasts, and chondrocytes [20]. One plausible explanation is that vitamin D reduces the production of inflammatory cytokines such as TNF-α and interleukin-1 beta [21,22], which are associated with OA and a more severe presentation.

TNF-α is a pro-inflammatory cytokine produced by chondrocytes, mononuclear cells, osteoblasts, and synovial tissue. It synergistically regulates the synthesis of extracellular matrix components by inhibiting anabolic activity and promoting the production of catabolic inflammatory factors [23,24]. TNF-α also stimulates the synthesis of proteolytic enzymes, such as metalloproteinases, which degrade cartilage matrix proteins [23,25]. Furthermore, it induces the production of other mediators, including interleukin-6 [25], which modulates an increase in the expression of metalloproteinases (MMPs) and a reduction in the expression of type II collagen [26].

Our findings suggest that TNF-α and IL-6 may be key mediators linking vitamin D deficiency to OA severity in older adults. Patients with vitamin D deficiency or insufficiency exhibited elevated serum levels of TNF-α and IL-6, which were not observed in control individuals without OA. These cytokines may contribute to joint damage and pain perception, making them potential therapeutic targets for OA [27]. However, additional studies are required to confirm this hypothesis.

Our study found that OA patients with low levels of vitamin D tended to have poorer functionality, in agreement with previous research [28]. While pain is the primary symptom contributing to functional disability in OA, studies examining the association between vitamin D levels and pain have yielded inconsistent results [29]. However, reduced vitamin D is associated with reduced pain in male patients with knee OA [11], indicating a potential sex-specific therapeutic approach that could be explored in future studies. 

Several limitations of this study should be acknowledged. The most significant limitation is the study design, which does not allow the establishment of a causal relationship. Indeed, the absence of follow-up data limits the assessment of the long-term impact of vitamin D deficiency on disease progression. Additionally, the lack of data on cytokine levels in synovial fluid was also a limitation, since such data could provide more direct evidence of local inflammation. However, it is noteworthy that not all patients had a surgical recommendation, and, taking into account that arthrocentesis in general conditions is an invasive procedure that could bring additional risks, we speculate that this approach could be considered in future studies, particularly in more severe patients who need surgical intervention. Therefore, cohort studies exploring several aspects, including diet and vitamin D supplementation history could help in validating these findings and better understanding the association between vitamin D deficiency and joint function.

Further, the primary hypothesis of this study was confirmed: reduced levels of vitamin D were associated with more severe osteoarthritis. Indeed, TNF-α and IL-6 seem to be the mediators involved on the deleterious effect of vitamin D deficiency in osteoarthritis. These findings may help to identify new therapeutic targets for OA and a potential beneficial effect of maintaining sufficient serum vitamin D for these patients.

Evidence suggests that deficiencies in both vitamin D and vitamin K increase the risk of OA, particularly as their levels decline with age [29,30]. In contrast, long-term effects of vitamin D supplementation were associated with improved WOMAC pain and function in OA patients compared to participants with insufficient vitamin D levels [31]. However, no studies to date have investigated the effects of combined supplementation with these vitamins for therapeutic purposes. Such an intervention could represent a promising strategy for preventing OA progression. Additionally, recommending routine vitamin D screening for elderly OA patients could also be considered.

## 5. Conclusions

Vitamin D deficiency is associated with increased pain, poorer functional status, and elevated serum levels of TNF-α and IL-6 in individuals with osteoarthritis. However, further research is required to elucidate the mechanisms through which vitamin D influences osteoarthritis and to evaluate the potential benefits of vitamin D supplementation for mitigating disease symptoms and progression.

## Figures and Tables

**Table 1:** Sociodemographic and clinical characteristics of the osteoarthritis subgroups.

Variables	Osteoarthritis Severity		*p* Value
	*Mild or Moderate*	*Severe*	
Age (median ± interquartile range)	68 ± 10	71 ± 9	0.07
BMI (median ± interquartile range)	27.7 ± 5.7	28.1 ± 5.5	0.74
Gender			
Female (n, percentage)	35 (67.3)	42 (80.8)	0.12
Male (n, percentage)	17 (32.7)	10 (19.2)	

**Table 2:** Vitamin D classification and severity of osteoarthritis.

Vitamin D Classification	Mild or Moderate	Severe	Total	*p* Value
Deficiency or Insufficiency	33 (43.4%)	43 (56.6%)	76 (100.0%)	0.04 *
Normal	19 (65.6%)	10 (34.5%)	29 (100.0%)	
Total	52 (49.5%)	53 (50.5%)	105 (100.0%)	

* Statistically significant, χ^2^ Test.

**Table 3:** Correlation between vitamin D levels and functional assessment of osteoarthritis.

Functionality	Correlation Index with Vitamin D Levels	
	rS	*p*
WOMAC Global	−0.25	0.01 *
WOMAC Pain	−0.21	0.03 *
WOMAC Stiffness	−0.17	0.09
WOMAC Function	−0.18	0.07

* Statistically significant, *p* <0.05.

## Data Availability

The original contributions presented in the study are included in article, further inquiries can be directed to the corresponding author.
